# Guidelines and practice on antipsychotics prescribing and physical health monitoring in children and young people: a cohort study using primary care data

**DOI:** 10.1136/bmjment-2024-301287

**Published:** 2025-07-08

**Authors:** Yana Vinogradova, Ruth H Jack, Vibhore Prasad, Carol Coupland, Richard Morriss, Chris Hollis

**Affiliations:** 1 Lifespan and Population Health, School of Medicine, University of Nottingham, Nottingham, UK; 2 Centre for Academic Primary Care, University of Nottingham, Nottingham, UK; 3 Department of Population Health Sciences, King’s College London, London, UK; 4 Mental Health and Neuroscience, School of Medicine, University of Nottingham, Nottingham, UK; 5 University of Nottingham, Nottingham, UK

**Keywords:** Child & adolescent psychiatry, Data Interpretation, Statistical

## Abstract

**Background:**

Antipsychotic treatments require physical health monitoring (PHM), especially among children and young people (CYP).

**Objective:**

For CYP aged 5-17, to investigate recorded indications for antipsychotics prescribing and first-treatment durations, and, for psychosis, bipolar disorder, autism spectrum disorder (ASD) and Tourette’s syndrome, recorded levels of PHM for CYP with antipsychotics prescriptions and those without.

**Methods:**

All CYP registered with QResearch English general practices between 2006 and 2021 were considered. To quantify PHM, 2158 CYP with antipsychotics prescriptions and 22 151 CYP with a condition but no prescriptions were followed for 2 years.

**Findings:**

47% (2363) of CYP with antipsychotics prescriptions had a recorded mental health condition of interest (of which 62% were ASD). 19% (921) had no relevant indication. For patients with ASD and Tourette syndrome, top quartiles for initial exposure to antipsychotics were >10 months. Recorded PHM was generally low, with over 50% of CYP showing no blood test during the 2-year follow-up.

**Conclusions:**

Coverage of best practice is uneven across the condition-related national CYP guidelines, and this requires improvement. However, we suspect some apparently poor adherence to best practice also derives from treatment complexities and associated data flows leading to gaps in the encoded general practice data. To audit more exactly clinical practice against guidelines, we propose qualitative studies, targeted to cover the full range of local circumstances, nationally.

**Clinical implications:**

General practices should be encouraged to prioritise encoding of all treatment data. Development of one central gold-standard set of recommendations for antipsychotics use could encourage better adherence levels across conditions.

**WHAT IS ALREADY KNOWN ON THIS TOPIC**

- Best practice guidelines from the UK National Institute for Health and Care Excellence (NICE) recommend referral to a specialist mental health service, such as the Child and Adolescent Mental Health Services or an Early Intervention in Psychosis service, for diagnosis, initial prescribing of antipsychotics and, for at least 12 months or until the patient’s condition has stabilised, subsequent physical health monitoring (PHM).
- Aftercare may then be transferred to primary care under shared-care arrangements.

**WHAT THIS STUDY ADDS**

- Focusing on the four mental health conditions explicitly linked to antipsychotics treatment in NICE guidelines, we report some concerning findings for recorded indications of prescribing, durations of exposure and postprescribing PHM.
- With respect to best-practice adherence, however, we discuss uneven guideline coverage and encoded data weaknesses consequent on treatment and data flow complexities, proposing streamlining of guidelines and, in the shorter term, an alternative research approach to audit, more reliably, adherence to/effects of national guidelines and policy initiatives.

**HOW THIS STUDY MIGHT AFFECT RESEARCH, PRACTICE OR POLICY**

- Measures to streamline national antipsychotics guidelines would facilitate access to best practice in all potential care settings, improving the scope of best-practice protection for patients.
- In the absence of mechanisms to ensure completeness of centrally encoded general practice records for these complex treatments, results from the research approach could more reliably audit actual adherence to best practice, nationally.

## Background

 Antipsychotics have side effects requiring specialist initial prescribing and regular physical health monitoring (PHM).^1^,^4^ Children and young people (CYP) are particularly affected by rapid weight gain and are more susceptible to metabolic syndrome.^5 6^ For CYP with first-episode psychosis or bipolar disorder, the UK National Institute for Health and Care Excellence (NICE) recommends that secondary care specialists prescribe antipsychotics and undertake/manage PHM for at least the first 12 months before transfer to primary care.^3 4^ General National Health Service (NHS) guidance^7^ and NICE guidelines recommend adequate monitoring should remain the responsibility of the prescriber, but it appears widely assumed that, post-transfer, PHM becomes the responsibility of primary care, despite weak links with specialists and decreasing primary care resources.^8^ Evidence suggests antipsychotics may be prescribed to about 11 000 CYP in England alone.^9^

Clearest recommendations for treatment of CYP appear in the NICE clinical guideline for psychosis and schizophrenia (CG155), which covers antipsychotics prescribing/initial PHM, transfer from specialist to primary care and subsequent shared care responsibilities.^3^ The bipolar disorder guideline is comprehensive, but combines information for adults and CYP.^4^ Although antipsychotics are not recommended for core treatment of autism spectrum disorder (ASD) or Tourette syndrome, the NICE guideline for under 19s with ASD^10^ and NICE recommendations for CYP with Tourette syndrome,^11^ both include information about off-label use. Here, however, advice on antipsychotics prescribing/aftercare is rudimentary, also making no reference to more detailed best practice guidelines.^10 11^ Although antipsychotics may be used more widely,^12^ NICE currently has no guidelines covering use for other mental health conditions affecting CYP.

A systematic review of interventions to improve antipsychotics PHM for CYP, mostly based on US insurance claims, revealed a large proportion of non-monitored CYP^13^ and guideline-practice shortfalls, with adherence higher for anthropometric measures, lower for laboratory tests.^14 15^ A small UK study has also found low levels of recorded metabolic monitoring of psychiatric patients,^16^ and the National Clinical Audit of Psychosis has produced evidence on best practice adherence focusing on general care within Early Intervention in Psychosis services.^17 18^ To our knowledge, no CYP-specific monitoring-adherence information from UK general practices is available.

### Objective

Our study is based on a sample of CYP, aged 5–17 years, representative of the English general population. It had three aims: (1) Describe trends/variations in encoded antipsychotics prescribing; (2) Describe encoded indications for and durations of such prescribing; and (3) Compare encoded records of PHM in primary care between CYP with antipsychotics prescriptions and those with mental health condition indications but no antipsychotics prescription. A parallel object of interest was data completeness—a possible issue for complex treatments involving separate care settings.

## Methods

The protocol has been published.^19^ We used the QResearch database, previously used for mental health drug-safety studies, containing data from English general practices linked to Hospital Episode Statistics (HES) admission and outpatient data.^20 21^ We identified an open cohort of patients aged 5–17 years, general-practice-registered between 1 January 2006 and 31 July 2021, and for at least 3 years before study entry.

All CYP were included for aim 1. We assessed recorded antipsychotics prescribing prevalence in general practices, both as a class and for the most commonly prescribed individual drugs (aripiprazole, olanzapine, prochlorperazine, quetiapine, risperidone). To estimate prescribing prevalence, the outcome was any antipsychotics prescription. For time trends, having at least one antipsychotic prescription during one calendar year was an outcome for that year.

For aim 2, we focused on incident antipsychotics prescribing in general practices, excluding those with prescriptions before study entry, examining incidence (and prevalence) rates of antipsychotics prescribing in boys and girls in two age groups: younger, aged 5–11 years; older, aged 12–17 years. We included: region; Townsend Deprivation Score quintile;^22^ and ethnicities listed in table 1. We used Poisson regression to calculate incidence rate ratios.

**Table 1:** Incidence rates and unadjusted rate ratios of prescribing antipsychotics in children aged 5–17 years in England, 2006–2021

Category	No of children with first Rx	Incidence rate per 10 000 person-years (95% CI)	Incidence rate ratio (95% CI)	Median (IQR) months of treatment
Total N	4985	3.81 (3.71 to 3.92)		2.4 (0.9 to 8.3)
Age group				
5–11 years	1063	1.45 (1.37 to 1.54)	Reference	2.6 (0.9 to 11.7)*
12–17 years	3922	6.81 (6.60 to 7.03)	5.15 (4.71 to 5.64)	2.4 (0.9 to 7.8)*
Sex				
Female	2088	3.33 (3.19 to 3.47)	Reference	2.1 (0.9 to 6.6)
Male	2897	4.25 (4.10 to 4.41)	1.28 (1.20 to 1.36)	2.7 (0.9 to 10.0)
Ethnicity				
White	2678	4.40 (4.23 to 4.57)	Reference	2.4 (0.9 to 8.7)
Asian	282	2.39 (2.13 to 2.69)	0.54 (0.48 to 0.62)	1.9 (0.9 to 6.8)
Black	108	2.52 (2.08 to 3.04)	0.57 (0.47 to 0.70)	1.9 (0.9 to 6.9)
Other	156	2.75 (2.35 to 3.22)	0.63 (0.53 to 0.74)	2.4 (0.9 to 8.0)
Unknown	1761	3.65 (3.49 to 3.83)	0.83 (0.77 to 0.90)	2.5 (0.9 to 8.4)
Townsend quintile†				
Affluent	1150	3.54 (3.35 to 3.76)	Reference	2.2 (0.9 to 7.6)
2	1093	3.88 (3.65 to 4.11)	1.09 (1.00 to 1.19)	2.8 (0.9 to 8.9)
3	1090	4.20 (3.95 to 4.45)	1.18 (1.08 to 1.29)	2.5 (0.9 to 8.8)
4	938	4.08 (3.82 to 4.34)	1.15 (1.04 to 1.27)	2.4 (0.9 to 8.5)
Deprived	675	3.27 (3.03 to 3.53)	0.92 (0.83 to 1.03)	2.3 (0.9 to 8.8)
Region (SHA)				
London	905	2.93 (2.75 to 3.13)	reference	2.6 (0.9 to 8.8)
East Midlands	154	4.70 (4.02 to 5.51)	1.61 (1.03 to 2.50)	3.5 (0.9 to 16.0)
East of England	195	3.57 (3.10 to 4.11)	1.22 (1.00 to 1.49)	2.5 (0.9 to 7.1)
North-East	142	3.52 (2.98 to 4.14)	1.20 (0.94 to 1.52)	3.1 (0.9 to 10.2)
North-West	786	3.05 (2.84 to 3.27)	1.04 (0.93 to 1.16)	2.1 (0.9 to 7.3)
South-Central	891	5.22 (4.89 to 5.57)	1.78 (1.56 to 2.03)	3.6 (0.9 to 10.4)
South-East	596	5.06 (4.67 to 5.48)	1.73 (1.48 to 2.01)	2.5 (0.9 to 8.5)
South-West	467	3.56 (3.26 to 3.90)	1.22 (1.03 to 1.43)	1.0 (0.9 to 6.7)
West Midlands	701	5.21 (4.84 to 5.61)	1.78 (1.54 to 2.06)	1.8 (0.9 to 7.1)
Yorkshire and Humber	148	2.48 (2.11 to 2.91)	0.85 (0.66 to 1.08)	1.7 (0.9 to 5.7)

* Age at the first prescription.

† For available data.

Rx, prescription; SHA, Strategic Health Authority.

Most recent indications recorded prior to the first prescription of any antipsychotic were extracted from practice records and linked HES admission and outpatient data. These included four mental health/neurological conditions of interest (psychosis/schizophrenia, bipolar disorder, ASD, Tourette syndrome), mental health symptoms (aggression/disruptive behaviour, self-harm), and mental health comorbidities (anxiety, attention-deficit/hyperactivity disorder (ADHD), depression, eating disorders, learning difficulties). Where multiple indications were recorded concurrently, we selected the most likely diagnostic prescribing indication for reporting, using the priority order presented in table 2. For antipsychotics as a class, we calculated for different indications the median (IQR) of durations of initial treatments in months, using prescription information and allowing a 90-day grace period between prescriptions.

**Table 2:** Numbers and proportions of patients with indications for the first antipsychotics prescription and duration of treatment, overall and by age at first prescription

	All ages	Median (IQR) months of treatment	5–11 years old	Median (IQR) months of treatment	12–17 years old	Median (IQR) months of treatment
MH condition recorded	2363 (47.4)	2.8 (0.9; 9.8)	582 (54.8)	2.8 (0.9; 12.1)	1781 (45.4)	2.8 (0.9; 9.1)
Psychosis*	545 (10.9)	2.6 (0.9; 7.3)	13 (1.2)	6.3 (1.1; 12.5)	532 (13.6)	2.6 (0.9; 7.2)
Bipolar disorder	67 (1.3)	2.2 (0.9; 5.5)	0	–	67 (1.7)	2.2 (0.9; 5.5)
Autism spectrum disorder	1463 (29.3)	2.9 (0.9; 10.6)	487 (45.8)	2.6 (0.9; 11.2)	976 (24.9)	3.0 (0.9; 10.6)
Tourette syndrome	288 (5.8)	3.0 (0.9; 13.1)	82 (7.7)	3.7 (0.9; 17.0)	206 (5.3)	2.9 (0.9; 10.0)
MH symptoms†—no MH condition recorded	596 (12.0)	2.2 (0.9; 6.8)	61 (5.7)	3.2 (0.9; 14.6)	535 (13.6)	2.2 (0.9; 6.5)
Only MH comorbidities‡ recorded	1105 (22.2)	2.8 (0.9; 8.8)	189 (17.8)	4.8 (0.9; 16.9)	916 (23.4)	2.5 (0.9; 7.5)
No relevant indication recorded	921 (18.5)	1.4 (0.9; 6.0)	231 (21.7)	0.9 (0.9; 5.3)	690 (17.6)	1.7 (0.9; 6.3)
All CYP with first prescriptions	4985	2.4 (0.9; 8.3)	1063	2.6 (0.9; 11.7)	3922	2.4 (0.9; 7.8)

* This covers psychosis and schizophrenia. 20 patients in this group were also diagnosed with bipolar disorder.

† MH symptoms include aggression/disruptive behaviour and self-harm.

‡ MH comorbidities include anxiety, attention-deficit/hyperactivity disorder, depression, eating disorders, learning difficulties.

CYP, children and young people; MH, mental health.

For aim 3, PHM in general practices, CYP with incident general practice antipsychotics prescriptions were compared with CYP with no encoded antipsychotic prescriptions but with a first record of a mental health condition. The participants were followed for 2 years from the first prescription or mental health condition record. PHM measures included are listed in table 3. To calculate exposure to antipsychotics during follow-up, we used all prescriptions within the 2-year period, calculating number of days from the prescription information. We also computed the number and proportion of patients with PHM measurements for both groups. In 2013, NICE issued updated antipsychotics treatment guidelines for CYP with psychosis/schizophrenia, refining recommendations for postprescribing monitoring and associated care responsibilities for secondary and primary care.^3^ We ran an additional analysis, restricted to 2013–2021, to identify postintervention differences.

**Table 3:** Number and proportion of patients with measurements during 2006–2021, over the first 2 years after the first prescription or diagnosis of mental health disorder, by disorder

	Total N	Proportion (%) of days on antipsychotics, median (IQR)	Heart N (%)	Anthropometrics N (%)	Lipids N (%)	Blood glucose N (%)	Prolactin N (%)
On antipsychotics							
Psychosis	133	48 (9; 90)	100 (75.2)	90 (67.7)	45 (33.8)	54 (40.6)	35 (26.3)
Bipolar	23	65 (8; 100)	18 (78.3)	18 (78.3)	11 (47.8)	13 (56.5)	9 (39.1)
ASD	751	47 (8; 95)	335 (44.6)	426 (56.7)	142 (18.9)	193 (25.7)	142 (18.9)
Tourette	178	35 (8; 84)	93 (52.2)	89 (50.0)	33 (18.5)	50 (28.1)	30 (16.9)
No coded MH condition	1073	33 (6; 85)	571 (53.2)	622 (58.0)	183 (17.1)	306 (28.5)	183 (17.1)
Overall	2158	38 (8; 88)	1117 (51.8)	1245 (57.7)	414 (19.2)	616 (28.5)	399 (18.5)
No antipsychotics							
Psychosis	2119	n/a	840 (39.6)	839 (39.6)	77 (3.6)	323 (15.2)	41 (1.9)
Bipolar	17	n/a	12 (70.6)	10 (58.8)	<5	<5	0
ASD	16 407	n/a	4475 (27.3)	5899 (36.0)	144 (0.9)	830 (5.1)	60 (0.4)
Tourette	3608	n/a	833 (23.1)	1131 (31.3)	27 (0.7)	194 (5.4)	9 (0.2)
Overall	21 151	n/a	6160 (27.8)	7879 (35.6)	n/r	n/r	110 (0.5)

Heart measurements include: blood pressure; pulse. Anthropometrics: weight, body mass index. Lipids: total cholesterol; HDL; LDL. Blood glucose: fasting glucose; HbA1C.

ASD, autism spectrum disorder; MH, mental health; n/a, not applicable; n/r, not reportable.

## Findings

### Recorded prevalence

QResearch contained, between 2006 and 2021, 2782 458 general-practice-registered CYP, aged 5–17 years, with at least 3 years of medical records before cohort entry. Of those, 5900 (0.21%) had one or more antipsychotic prescription(s) during the period (online supplemental eFigure 1, flow chart). Online supplemental eTable 1 presents demographics for CYP in the general population and those with antipsychotic prescriptions.

**Figure 1:**
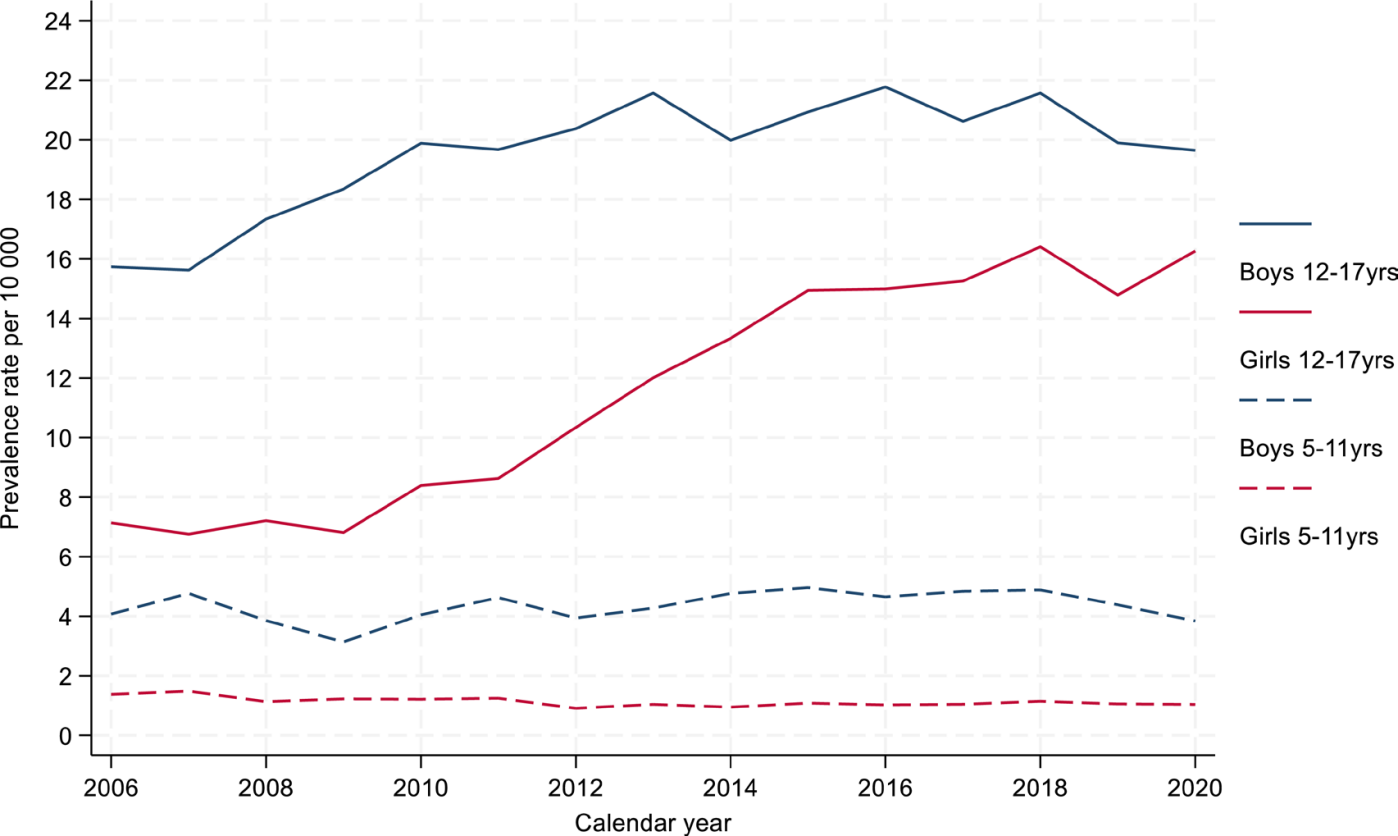
Prevalence rate for antipsychotic prescribing by gender and age by calendar year in children aged 5–17 years.

For annual prevalence of any antipsychotics use, we found, overall, a 45% increase in prescribing, rising from 6.9 per 10 000 individuals in 2006 to 10.0 in 2018 (online supplemental eFigure 2). Patients aged 12–17 years had higher rates of antipsychotics prescriptions (between 11.5 and 18.0 per 10 000) than those aged 5–11 years (between 2.2 and 3.2) (online supplemental eFigure 2). More boys were prescribed, but the rate of prescribing increase among older girls was almost twice that in the equivalent male group, respectively, 16.4 against 7.2 per 10 000 (2.3 times) and 21.6 against 17.3 (1.2 times) (figure 1). Atypical antipsychotics were more commonly prescribed, with risperidone the most used. However, from 2013 to 2020 risperidone use fell (from 5.8 to 4.1 per 10 000), while aripiprazole use rose over the study period (from 0.3 to 3.1) (online supplemental eFigure 3).

### Recorded incidence rates

Over the study period, 4985 CYP had their first antipsychotics prescription. Table 1 shows incidence rates, rate ratios and median duration of initial treatments for different demographic groups. Older patients were much more likely to start treatment, and, overall, boys were more likely than girls. White ethnicity CYP were more likely to start than other ethnicities. There were some variations between levels of deprivation and different regions but not consistently over the study period. Median duration of first treatment was 2.4 months (IQR 0.9; 8.3). Boys had longer initial exposure than girls (median 2.7 vs 2.1 months) but no other notable variations emerged (table 1). Incidence rates increased over the study period from 2.8 per 1000 person-years in 2007 to 4.8 in 2018, again most pronouncedly in older girls (online supplemental eFigure 4).

### Recorded indications and durations

Table 2 breaks down most recently recorded indications for the first antipsychotic prescription, giving median durations (IQRs) for initial exposures, overall and by age group. 47.4% of patients had a mental health condition of interest: psychosis 10.9%, bipolar disorder 1.3%, ASD 29.3%, Tourette syndrome 5.8%. 12.0% had no such records but a mental health symptom record (aggressive/disruptive behaviour or self-harm). 22.2% had neither of these but a mental health comorbidity record (anxiety, ADHD, depression, eating disorders, learning difficulties). 18.5% had no relevant indications.

Age breakdowns showed sizeable differences between the conditions: psychosis (younger, 1.2%; older 13.6%) and bipolar disorder (younger, 0%; older, 1.7%) versus ASD (younger 45.8%; older 24.9%) and Tourette syndrome (younger 7.7%; older 5.3%). 5.7% of younger and 13.6% of the older patients had only symptom indication(s) while 17.8% of the younger and 23.4% of the older had only mental health comorbidity indication(s). 21.7% of the younger and 17.6% of the older patients had no recorded indications.

For CYP with mental health conditions, the median duration of initial antipsychotics treatment was 2.8 months, for all age groups (table 2). For older CYP, median durations differed relatively slightly by indications for prescribing, shorter for psychosis (2.6 months) and bipolar disorder (2.2 months), longer for ASD (2.9 months) and Tourette syndrome (3.0 months), but top quartiles for ASD (10.6 months) and Tourette syndrome (13.1 months) were notably higher than for psychosis (7.3 months) or bipolar disorder (5.5 months). For younger CYP, only the ASD and Tourette syndrome groups had sufficient patients for estimation, with median duration for ASD slightly lower than the overall and for Tourette syndrome slightly higher, but the top quartiles were again high (11.2 months and 17.0 months, respectively).

Among the IQR figures for CYP with no recorded mental health condition of interest but with symptom and/or mental health comorbidity record(s), there were some individual symptoms and comorbidities also with relatively high top quartiles. Most notably, these included self-harm and eating disorders among younger CYP, and, more generally, ADHD and learning difficulties, the latter both frequently linked to patients with ASD (online supplemental eTable 3).

### Follow-up analysis: recorded prescribing

A population-representative sample of CYP had at least 2 years of follow-up data—2158 with antipsychotics prescriptions; 22 151 with a recorded mental health condition but no prescriptions (online supplemental eTable 4). Overall, CYP prescribed antipsychotics had a median of 37% (IQR: 8%; 88%) of days covered by prescriptions during the 2-year follow-up period. No clear condition-dependent pattern was evident in the median proportion of days (IQR) for CYP on antipsychotics: 65% (8%; 100%) for bipolar disorder; 48% (9%; 90%) for psychosis; 47% (8%; 95%) for ASD; 35% (8%; 84%) for Tourette syndrome; 33% (6%; 85%) for patients with no relevant mental health condition encoded. The commonly high treatment-duration levels of the top quartiles were, however, noteworthy.

### Follow-up groups: recorded monitoring

Although low bipolar disorder numbers made some estimates less certain (some unreportable), the proportions of patients with monitoring records did vary by mental health condition. Of patients prescribed antipsychotics, those with indications for psychosis or bipolar disorder were more likely to have monitoring records than patients with indications for ASD or Tourette syndrome. This held for simple measurements like anthropometrics (weight/body mass index): psychosis (68%) and bipolar disorder (78%); ASD (57%) and Tourette syndrome (50%). It also held for measurements requiring tests like blood glucose monitoring: psychosis (41%) and bipolar disorder (57%); ASD (26%) and Tourette syndrome (28%). Among patients not on antipsychotics, this pattern of condition-related difference was also evident. Anthropometric proportions were: psychosis (40%) and bipolar disorder (59%); ASD (36%) and Tourette syndrome (31%). Blood glucose proportions were: psychosis (15%) and bipolar disorder (unreportable); ASD (5%) and Tourette syndrome (5%) (table 3).

Weight, height and blood pressure were the most recorded measures in both groups; respectively, 56%, 51% and 48% of CYP on antipsychotics, 34%, 31% and 18% of CYP with no antipsychotics. For >50% of CYP over the 2-year follow-up, these measures were recorded twice in the group prescribed antipsychotics compared with once for those with no antipsychotic records. Less recorded overall, lipids were recorded more within the antipsychotics group: between 13% and 19% (measure-dependent) of those prescribed antipsychotics compared with 1% or less of those not. Fasting glucose and glycated hemoglobin were similarly more recorded for CYP with antipsychotic prescriptions (24% and 11%, respectively) than those without (4% and 3%). For >50% of CYP, these measures were recorded only once (online supplemental eTable 5).

### Additional analysis

The post-2013 NICE psychosis/schizophrenia updates analysis included 14 989 CYP with mental health disorder but no antipsychotics records and 1285 with antipsychotics records, all with 2 years of follow-up data. Proportions of CYP with recorded measures were found to be only marginally higher than in the main analysis. Descriptive statistics for demographics, baseline information for selected patients and monitoring information are in supplementary data (online supplemental eTables 6–8).

## Discussion

### Principal findings

There is considerable variation across mental health conditions in the comprehensiveness of NICE recommendations for clinicians considering antipsychotic treatments for CYP. Best-practice splits in treatment management responsibility—diagnosis, initial prescribing, and a significant period of initial PHM monitoring and treatment stabilisation in secondary care, before transfer to primary care for management of repeat prescribing and ongoing PHM. These complex processes may well adversely affect the completeness of encoded general practitioner records (see Strengths and weaknesses, below). The first could result in deviations from best practice, and the second could incorrectly evidence such deviations.

Prescription data are core to general practice record-keeping, so are normally reliable. Our results confirmed expected prevalence and incidence rises in antipsychotics prescribing to CYP—more in adolescence than childhood, greatest among adolescent girls, mirroring observed increases in mental health disorders within these groups.^23^ However, even here our results may underestimate reality because of treatment and monitoring in secondary care missing from encoded general practice records.

Under half of CYP prescribed antipsychotics had recorded indications for a relevant mental health condition. Of these, three quarters were for ASD or Tourette syndrome—conditions for which antipsychotics prescribing to CYP is off-label and should be limited, but where NICE guidance is weakest. A third of CYP had indications only for mental health symptoms and/or comorbidities. One in five had no relevant indications, confirming our concern about gaps in encoded data.

For CYP with indications of ASD or Tourette syndrome, we found recorded exposure durations of over 10 months for the quarter of patients with longest exposures (17 months among the younger Tourette syndrome group). Among CYP with only mental health comorbidity/ies recorded, the same was true for ADHD and learning difficulties (symptoms associated with ASD). Younger CYP exhibiting self-harm symptoms also showed relatively high 10-month exposure durations.

The follow-up of CYP with encoded indications for a relevant mental health condition showed relatively high proportions of days exposed but lower monitoring levels for the ASD and Tourette syndrome groups, appearing to confirm again concerns about unequal NICE best practice coverage for these conditions. However, we suspect the generally low PHM levels, and lower proportions of patients with records for laboratory measurements than for basic measures, possibly reflect more the incomplete encoding of relevant data held in other forms within general practices.

### Strengths and weaknesses

QResearch database is one of the leading NHS national-level primary care data sources, used internationally for epidemiological research. All of its encoded general practice data are linked to Child and Adolescent Mental Health Services, hospital admissions and outpatient appointments data.

However, there is no national information source which fully integrates treatment data from general practices data with treatment records from secondary care and specialist community care groups. Linked hospital information was limited to diagnosis information, with no data for prescribed medications, PHM or inpatient treatments, and its completeness possibly depends on individual patient care histories and local policy/clinical decisions. Information from specialised care groups, like Early Intervention in Psychosis or those that manage treatments for some CYP diagnosed with ASD and Tourette syndrome treatments is not linked.

Data from unlinked care settings and data missing from hospital linkages are normally transferred to general practices, unencoded, at treatment handovers. However, the frequency of handovers and the completeness of included information possibly vary. All data received, including PHM information from laboratories or other external assessments, need to be encoded by general practices for it to become integrated with internally generated treatment records. Despite improving levels of recording in UK general practices,^24^ our analysis for 2013–2021 showed only marginally higher PHM levels after the 2013 psychosis guideline reissue,^3^ so a—possibly *the*—crucial unknown is the extent and consistency, across practices, of such encoding.

Gaps in the encoded data available to researchers could, therefore, include prescription records, associated indications for prescribing and PHM records. Apart from the direct effects of data loss, such gaps would also increase uncertainty about accurate identification of subgroups of patients, for example, patients prescribed antipsychotics/patients not prescribed.

### Comparisons with other studies

A Clinical Practice Research Datalink (CPRD)-Aurum (similar to QResearch) study investigated rates of and indications for antipsychotics prescribing to CYP in England between 2000 and 2019 without investigating associated PHM.^9^ It had a slightly extended upper age limit (19 years) but apparently lacked access to hospital and outpatient data. Overall findings on prevalent and incident prescribing were similar, but their proportions of indications for our four mental health conditions of interest were even lower, especially for ASD. Our linkages to hospital data could have led to some initial general practice indications being overridden by later hospital indications, possibly explaining our mostly higher proportion of mental health condition indications—their sole higher proportion, for bipolar disorder, being attributable to their higher upper age limit.

All studies of PHM in a systematic review of papers from differing countries/environments (none UK-based) showed higher rates for basic monitoring (height, weight, body mass index) than for laboratory tests (blood, lipid and liver).^13^ These studies did generally demonstrate some improvements after interventions/new guidelines, while we found no differences after the 2013 NICE psychosis guideline reissue.^3^

An NHS-supported project (Stopping Over Medication of People (STOMP)) launched in 2016 to reduce inappropriate use of psychotropic drugs when managing behaviour was deemed challenging (so one focus was treatments of patients with ASD). A parallel CYP-oriented STOMP-STAMP (Supporting Treatment and Appropriate Medication in Pediatrics) programme was launched in late 2018.^25 26^ A study followed, measuring early project outcomes and testing the assessment methodology, and also using a source of encoded general practice records.^27^ Their data source was not one of the better known and the study was more methodology-focused than results-focused, so, while the researchers expressed concerns about future stability/growth of their data source, they were silent about data completeness.^27^

### Unanswered questions and future research

Evidence from our study and the CPRD Aurum study^9^ suggest some ‘normalisation’ of antipsychotics use in treatments of CYP most clearly with respect to ASD and Tourette syndrome, both of which affect younger CYP. However, sections within relevant NICE guidelines covering antipsychotics prescribing and/or subsequent PHM remain rudimentary.^9 11^ Antipsychotics also appear to have been used for the treatment of mental health symptoms or comorbidities unsupported by any NICE clinical guidelines. Possibly as the result of STOMP-STAMP, some additional guidelines for patients with learning difficulties have emerged (NG93, NG54, NG11), the last of which does reference the ‘gold standard’ CG155 psychosis guideline, but with different PHM scheduling for expected short treatment durations.^28^,^30^

However, the low levels of appropriate indications for antipsychotics prescribing to CYP in the two studies, echoed in the low levels of recorded PHM that we found, raise concerns about the completeness of encoded general practice records, specifically with respect to these complex treatments. The issue is most likely structural and environmental—responsibility splits for prescribing and PHM at different stages of treatment, multiple information sources, formats, data flows and destinations—all against a backdrop of stretched resources, and with no defined process/assigned responsibility to encourage ultimate completeness of encoded general practice records. Many general practitioners probably have access to fuller information in other formats not available for national audits or research. Until this can be addressed, any adherence auditing using only encoded sources will be questionable.

The CPRD study proposed ‘a national audit of existing prescribing practices’ to check adherence to recommendations and identify guidance-implementation gaps.^9^ However, the National Clinical Audit of Psychosis shows the level of effort required even when restricted to one service and avoiding complexities such as prescribing indications and timings of PHM.^17 18^ A more detailed national audit of adherence to NICE best practice, including all relevant care services, is almost certainly infeasible because of current barriers to full data access. Instead, we would suggest qualitative research targeted at a range of nationally representative care environments to unpick the realities of practice detail. This should:

- Audit prescribing and PHM practice against NICE ‘gold standard’ (CG155) recommendations for antipsychotic treatments of CYP, the autdit include indication(s) for prescribing, frequency and completeness of PHM (in both secondary and primary care), secondary to primary care handovers, and the sharing of posthandover aftercare and any other subsequent primary-secondary care contacts;
- Investigate associated data flows and encoding practice to and within general practices, including all aspects of treatment and aftercare, and the final destination and format of transmitted information.

Limiting inclusion to young people (perhaps extending the CYP upper age limit to better cover bipolar disorder) would contain costs but retain focus on indications for which antipsychotics are generally not considered core treatments. Such research could deliver information useful also to the STOMP and STOMP-STAMP initiative.

### Clinical implications

In the short term, clinicians involved in any antipsychotics treatment of CYP should be alerted to the existence of CG155 and NG11 and encouraged to follow these newer/more complete best practice recommendations. General practices should be encouraged to prioritise the encoding of treatment information relating to mental health treatments.

In the longer term, a centralised, condition-independent version of guidelines for antipsychotic treatments of CYP, based on the current CG155 (for psychosis and schizophrenia), could usefully be developed. Taking into account resource issues, this could introduce baseline requirements for handovers of care and continuing care, for transmission of data and for ultimate encoding of data by general practices. It would both better disseminate best practice than the current proliferation of different sources, and facilitate research—including more accurate national audits of adherence.

## Supplementary material

10.1136/bmjment-2024-301287 online supplemental file 1

## Data Availability

Data are available upon reasonable request. No data are available.
