# Supplementary material for: Guidelines and practice on antipsychotics prescribing and physical health monitoring in children and young people: a cohort study using primary care data
Source: BMJ Ment Health. 2025 Jul 8;28(1):e301287. doi: 10.1136/bmjment-2024-301287 (PMC12243589; doi:10.1136/bmjment-2024-301287)
Supplement: online supplemental file 1 [file bmjment-28-1-s001.docx]

# Supplementary data

# Guidelines and practice on antipsychotics prescribing and physical health monitoring in children and young people: a cohort study using primary care data

Yana Vinogradova, Ruth H Jack, Vibhore Prasad, Carol Coupland, Richard Morriss, Chris Hollis

## Supplementary tables

[eTable 1 Demographic characteristics for children prescribed and not prescribed antipsychotics 3](#_Toc197501332)

[eTable 2 Number and proportion of patients with a first prescription for different antipsychotics during the study period 5](#_Toc197501333)

[eTable 3 Duration of treatment, overall and by age at first prescription of antipsychotic in patients with recorded mental health symptoms and mental health comorbidities. 6](#_Toc197501334)

[eTable 4 Descriptive statistics for two cohorts at the time of first antipsychotic prescription or first records of mental health disorder, **2006-2021**, all patients were followed up for 2 years, numbers(%) 7](#_Toc197501335)

[eTable 5 Number and proportion of patients with measurements and median (interquartile range) of number of records for patients with measurements, number of children first diagnosed diabetes, **2006-2021**, over the first two years after the first prescription or diagnosis of mental health problem 8](#_Toc197501336)

[eTable 6 Descriptive statistics for two cohorts at the time of first antipsychotic prescription or first records of mental health disorder, **2013-2021**, all patients were followed up for 2 years, numbers(%) 9](#_Toc197501337)

[eTable 7 Number and proportion of patients with measurements and median (interquartile range) of number of records for patients with measurements, number of children first diagnosed diabetes, **2013-2021**, over the first two years after the first prescription or diagnosis of mental health problem 10](#_Toc197501338)

[eTable 8 Number and proportion of patients with measurements **2013-2021**, over the first two years after the first prescription or diagnosis of mental health disorder, by metal health disorder 10](#_Toc197501339)

## Supplementary figures

[eFigure 1 Flow-chart for included individuals 2](#_Toc155537599)

[eFigure 2 Prescribing by age group 4](#_Toc155537600)

[eFigure 3 Prescribing by individual antipsychotic drug 4](#_Toc155537601)

[eFigure 4 Incidence rate by age and gender 5](#_Toc155537602)

eFigure 1 Flow-chart for included individuals


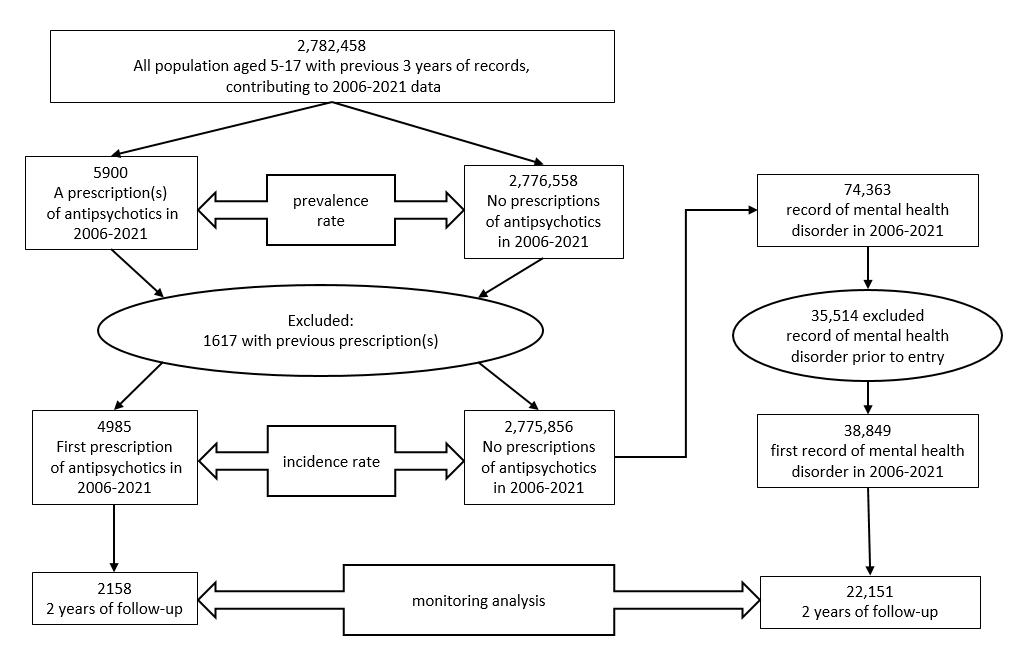


eTable 1 Demographic characteristics for children prescribed and not prescribed antipsychotics

| category | N (%) for CYP with antipsychotic prescriptions | Proportion with antipsychotic prescriptions | N (%) for CYP general population excluding children with antipsychotic prescriptions |
| --- | --- | --- | --- |
| Overall | 5,900 | 0.21% | 2,776,558 |
| **Sex** |  |  |  |
| Female | 2306 (39.1) | 0.17% | 1,335,340 (48.1) |
| Male | 3594 (60.9) | 0.25% | 1,441,218 (51.9) |
| **Ethnicity** |  |  |  |
| White | 3197 (54.2) | 0.24% | 1,307,394 (47.1) |
| Asian | 332 (5.6) | 0.13% | 252915 (9.1) |
| Black | 132 (2.2) | 0.13% | 99648 (3.6) |
| Other | 199 (3.4) | 0.14% | 143518 (5.2) |
| Unknown | 2039 (34.6) | 0.21% | 973083 (35.0) |
| **Townsend quintile** |  |  |  |
| Affluent | 1383 (23.4) | 0.22% | 631069 (22.7) |
| 2 | 1309 (22.2) | 0.23% | 581013 (20.9) |
| 3 | 1281 (21.7) | 0.23% | 561686 (20.2) |
| 4 | 1100 (18.6) | 0.21% | 518958 (18.7) |
| Deprived | 784 (13.3) | 0.17% | 470252 (16.9) |
| Unknown | 43 (0.7) | 0.32% | 13580 (0.5) |
| **Region (SHA)** |  |  |  |
| London | 171 (2.9) | 0.15% | 61607 (2.2) |
| East Midlands | 221 (3.8) | 0.28% | 109775 (4.0) |
| East of England | 1058 (17.9) | 0.20% | 711766 (25.6) |
| North East | 167 (2.8) | 0.23% | 73091 (2.6) |
| North West | 930 (15.8) | 0.18% | 529141 (19.1) |
| South Central | 1049 (17.8) | 0.29% | 361425 (13.0) |
| South East | 708 (12.0) | 0.27% | 264987 (9.5) |
| South West | 538 (9.1) | 0.21% | 259918 (9.4) |
| West Midlands | 858 (14.5) | 0.29% | 291396 (10.5) |
| Yorkshire & Humber | 200 (3.4) | 0.18% | 113452 (4.1) |

eFigure 2 Prescribing by age group

eFigure 3 Prescribing by individual antipsychotic drug

eFigure 4 Incidence rate by age and gender

eTable 2 Number and proportion of patients with a first prescription for different antipsychotics during the study period

| Drug | N (%) |
| --- | --- |
| Aripiprazole | 868 (17.4) |
| Olanzapine | 523 (10.5) |
| Quetiapine | 676 (13.6) |
| Risperidone | 2453 (49.2) |
| Chlorpromazine | 65 (1.3) |
| Haloperidol | 79 (1.6) |
| Other | 287 (5.8) |
| Conflicting | 34 (0.7) |
| Total | 4985 (100) |

eTable 3 Duration of treatment, overall and by age at first prescription of antipsychotic in patients with recorded mental health symptoms and mental health comorbidities.

|  | All ages | Median (interquartile range) months of treatment | 5-11 years old | Median (interquartile range) months of treatment | 12-17 years old | Median (interquartile range) months of treatment |
| --- | --- | --- | --- | --- | --- | --- |
| **Mental health Symptoms*** | 1068 (21.4) | 2.0 (0.9; 6.8) | 143 (13.5) | 1.4 (0.9; 8.7) | 925 (23.6) | 2.1 (0.9; 6.7) |
| Aggression/ disruptive behaviour | 547 (11.0) | 2.5 (0.9; 9.3) | 135 (12.7) | 1.4 (0.9; 8.7) | 412 (10.5) | 2.8 (0.9; 9.3) |
| Self-harm | 572 (11.5) | 1.9 (0.9; 5.9) | 13 (1.2) | 4.1 (0.9; 10.0) | 559 (14.3) | 1.9 (0.9; 5.9) |
| **Mental health comorbidities*** | 2735 (54.9) | 2.8 (0.9; 8.9) | 507 (47.7) | 3.9 (0.9; 13.3) | 2228 (56.8) | 2.6 (0.9; 8.0) |
| Anxiety | 845 (17.0) | 1.7 (0.9; 6.2) | 76 (7.1) | 2.4 (0.9; 6.9) | 769 (19.6) | 1.7 (0.9; 6.1) |
| Attention deficit/ hyperactivity disorder | 1045 (21.0) | 3.7 (0.9; 12.8) | 369 (34.7) | 3.9 (0.9; 15.2) | 676 (17.2) | 3.6 (0.9; 11.4) |
| Depression | 783 (15.7) | 2.2 (0.9; 6.2) | 5 (0.5) | 6.0 (5.1; 7.8) | 778 (19.8) | 2.2 (0.9; 6.2) |
| Eating disorders | 295 (5.9) | 2.5 (0.9; 6.6) | 6 (0.6) | 2.0 (0.9; 11.7) | 289 (7.4) | 2.5 (0.9; 6.6) |
| Learning difficulties | 347 (7.0) | 3.8 (0.9; 12.5) | 122 (11.5) | 3.9 (0.9; 10.2) | 225 (5.7) | 3.6 (0.9; 13.6) |

*) A patient may have more than 1 symptom or comorbidity recorded

eTable 4 Descriptive statistics for two cohorts at the time of first antipsychotic prescription or first records of mental health disorder, **2006-2021**, all patients were followed up for 2 years, numbers(%)

|  | Mental health disorders or on antipsychotics (col%) | On antipsychotics  (row%) | No antipsychotics (row%) |
| --- | --- | --- | --- |
| **Total N** | 24309 (100) | 2158 (8.9) | 22151 (91.1) |
| **Age at the start** |  |  |  |
| 5 to 11 years | 16473 (67.8) | 773 (4.7) | 15700 (95.3) |
| 12 to 17 years | 7836 (32.2) | 1385 (17.7) | 6451 (82.3) |
| Mean (SD) | 22 (0.1) | 12 (56.0) | 10 (44.0) |
| **Sex** |  |  |  |
| Female | 6459 (26.6) | 729 (11.3) | 5730 (88.7) |
| Male | 17850 (73.4) | 1429 (8.0) | 16421 (92.0) |
| **Ethnicity** |  |  |  |
| White | 13532 (55.7) | 1200 (8.9) | 12332 (91.1) |
| Asian | 1318 (5.4) | 110 (8.3) | 1208 (91.7) |
| Black | 562 (2.3) | 41 (7.3) | 521 (92.7) |
| Other | 883 (3.6) | 50 (5.7) | 833 (94.3) |
| Unknown | 8014 (33.0) | 757 (9.4) | 7257 (90.6) |
| **Townsend quintile** |  |  |  |
| Affluent | 5584 (23.0) | 476 (8.5) | 5108 (91.5) |
| 2 | 5540 (22.8) | 480 (8.7) | 5060 (91.3) |
| 3 | 5317 (21.9) | 500 (9.4) | 4817 (90.6) |
| 4 | 4549 (18.7) | 406 (8.9) | 4143 (91.1) |
| Deprived | 3210 (13.2) | 281 (8.8) | 2929 (91.2) |
| Unknown | 109 (0.4) | 15 (13.8) | 94 (86.2) |
| **Region (SHA)** |  |  |  |
| London | 4331 (17.8) | 379 (8.8) | 3952 (91.2) |
| East Midlands | 678 (2.8) | 76 (11.2) | 602 (88.8) |
| East of England | 1072 (4.4) | 78 (7.3) | 994 (92.7) |
| North East | 789 (3.2) | 84 (10.6) | 705 (89.4) |
| North West | 4685 (19.3) | 310 (6.6) | 4375 (93.4) |
| South Central | 3902 (16.1) | 416 (10.7) | 3486 (89.3) |
| South East | 2803 (11.5) | 230 (8.2) | 2573 (91.8) |
| South West | 2254 (9.3) | 203 (9.0) | 2051 (91.0) |
| West Midlands | 2858 (11.8) | 310 (10.8) | 2548 (89.2) |
| Yorkshire & Humber | 937 (3.9) | 72 (7.7) | 865 (92.3) |
| **Mental health conditions** |  |  |  |
| Severe psychosis | 2252 (9.3) | 133 (5.9) | 2119 (94.1) |
| Bipolar disorder | 40 (0.2) | 23 (57.5) | 17 (42.5) |
| Autism spectrum disorder | 17158 (70.6) | 751 (4.4) | 16407 (95.6) |
| Tourette syndrome | 3786 (15.6) | 178 (4.7) | 3608 (95.3) |
| **Mental health symptoms*** |  |  |  |
| Aggression/ disruptive behaviour | 1314 (5.4) | 280 (21.3) | 1034 (78.7) |
| Self harm | 378 (1.6) | 136 (36.0) | 242 (64.0) |
| **Mental health comorbidities*** |  |  |  |
| Anxiety | 1174 (4.8) | 248 (21.1) | 926 (78.9) |
| Attention deficit hyperactivity disorder | 2846 (11.7) | 622 (21.9) | 2224 (78.1) |
| Depression | 1313 (5.4) | 164 (12.5) | 1149 (87.5) |
| Eating disorder | 174 (0.7) | 96 (55.2) | 78 (44.8) |
| Learning difficulties | 1446 (5.9) | 179 (12.4) | 1267 (87.6) |
| **Diabetes (any type)** | 74 (0.3) | 7 (9.5) | 67 (90.5) |

*) A patient may have more than 1 symptom or comorbidity recorded

eTable 5 Number and proportion of patients with measurements and median (interquartile range) of number of records for patients with measurements, number of children first diagnosed diabetes, **2006-2021**, over the first two years after the first prescription or diagnosis of mental health problem

|  | Monitoring period 2006-2021 | | | |
| --- | --- | --- | --- | --- |
|  | No antipsychotics | | On antipsychotics | |
|  | N (%) | Median (IQR) | N (%) | Median (IQR) |
| Total | 22151 |  | 2158 |  |
| Proportion of days on the drugs |  | N/A |  | 37 (8; 88) |
| Systolic BP | 3978 (18.0) | 1 (1; 3) | 1039 (48.1) | 2 (1; 3) |
| Diastolic BP | 3972 (17.9) | 1 (1; 3) | 1037 (48.1) | 2 (1; 3) |
| Pulse | 4260 (19.2) | 1 (1; 2) | 650 (30.1) | 1 (1; 2) |
| Height | 6810 (30.7) | 1 (1; 2) | 1089 (50.5) | 2 (1; 3) |
| Weight | 7579 (34.2) | 1 (1; 2) | 1215 (56.3) | 2 (1; 3) |
| BMI | 4482 (20.2) | 1 (1; 2) | 843 (39.1) | 1 (1; 2) |
| Total cholesterol | 249 (1.1) | 1 (1; 1) | 413 (19.1) | 1 (1; 2) |
| HDL | 223 (1.0) | 1 (1; 1) | 378 (17.5) | 1 (1; 2) |
| LDL | 165 (0.7) | 1 (1; 1) | 288 (13.3) | 1 (1; 2) |
| Glucose | 980 (4.4) | 1 (1; 1) | 518 (24.0) | 1 (1; 2) |
| HbA1c | 562 (2.5) | 1 (1; 1) | 238 (11.0) | 1 (1; 1) |
| Prolactin | 110 (0.5) | 1 (1; 1) | 399 (18.5) | 1 (1; 2) |
| Diagnosed with diabetes | 30 (0.1) |  | 12 (0.6) |  |

eTable 6 Descriptive statistics for two cohorts at the time of first antipsychotic prescription or first records of mental health disorder, **2013-2021**, all patients were followed up for 2 years, numbers(%)

|  | Mental health disorders or on antipsychotics (col%) | On antipsychotics  (row%) | No antipsychotics (row%) |
| --- | --- | --- | --- |
| **Total N** | 16274 (100) | 1285 (7.9) | 14989 (92.1) |
| **Age at the start** |  |  |  |
| 5 to 11 years | 11016 (67.7) | 444 (4.0) | 10572 (96.0) |
| 12 to 17 years | 5258 (32.3) | 841 (16.0) | 4417 (84.0) |
| Mean (SD) | 22 (0.1) | 12 (56.2) | 10 (43.8) |
| **Sex** |  |  |  |
| Female | 4710 (28.9) | 485 (10.3) | 4225 (89.7) |
| Male | 11564 (71.1) | 800 (6.9) | 10764 (93.1) |
| **Ethnicity** |  |  |  |
| White | 9929 (61.0) | 765 (7.7) | 9164 (92.3) |
| Asian | 962 (5.9) | 69 (7.2) | 893 (92.8) |
| Black | 406 (2.5) | 32 (7.9) | 374 (92.1) |
| Other | 678 (4.2) | 30 (4.4) | 648 (95.6) |
| Unknown | 4299 (26.4) | 389 (9.0) | 3910 (91.0) |
| **Townsend quintile** |  |  |  |
| Affluent | 3648 (22.4) | 303 (8.3) | 3345 (91.7) |
| 2 | 3555 (21.8) | 270 (7.6) | 3285 (92.4) |
| 3 | 3604 (22.1) | 301 (8.4) | 3303 (91.6) |
| 4 | 3168 (19.5) | 239 (7.5) | 2929 (92.5) |
| Deprived | 2214 (13.6) | 164 (7.4) | 2050 (92.6) |
| Unknown | 85 (0.5) | 8 (9.4) | 77 (90.6) |
| **Region (SHA)** |  |  |  |
| London | 2912 (17.9) | 237 (8.1) | 2675 (91.9) |
| East Midlands | 432 (2.7) | 43 (10.0) | 389 (90.0) |
| East of England | 756 (4.6) | 39 (5.2) | 717 (94.8) |
| North East | 538 (3.3) | 32 (5.9) | 506 (94.1) |
| North West | 3312 (20.4) | 190 (5.7) | 3122 (94.3) |
| South Central | 2480 (15.2) | 254 (10.2) | 2226 (89.8) |
| South East | 1872 (11.5) | 147 (7.9) | 1725 (92.1) |
| South West | 1485 (9.1) | 108 (7.3) | 1377 (92.7) |
| West Midlands | 1881 (11.6) | 206 (11.0) | 1675 (89.0) |
| Yorkshire & Humber | 606 (3.7) | 29 (4.8) | 577 (95.2) |
| **Mental health disorders** |  |  |  |
| Severe psychosis | 1529 (9.4) | 77 (5.0) | 1452 (95.0) |
| Bipolar disorder | 19 (0.1) | 11 (57.9) | 8 (42.1) |
| Autism spectrum disorder | 11807 (72.6) | 476 (4.0) | 11331 (96.0) |
| Tourette syndrome | 2292 (14.1) | 94 (4.1) | 2198 (95.9) |
| **Symptoms*** |  |  |  |
| Aggression/ disruptive behaviour | 775 (4.8) | 148 (19.1) | 627 (80.9) |
| Self harm | 314 (1.9) | 113 (36.0) | 201 (64.0) |
| **Comorbidities*** |  |  |  |
| Anxiety | 976 (6.0) | 190 (19.5) | 786 (80.5) |
| Attention deficit hyperactivity disorder | 1928 (11.8) | 341 (17.7) | 1587 (82.3) |
| Depression | 931 (5.7) | 122 (13.1) | 809 (86.9) |
| Eating disorder | 134 (0.8) | 81 (60.4) | 53 (39.6) |
| Learning difficulties | 927 (5.7) | 90 (9.7) | 837 (90.3) |
| **Diabetes (any type)** | 61 (0.3) | 7 (11.5) | 54 (88.5) |

*) A patient may have more than 1 symptom or comorbidity recorded

eTable 7 Number and proportion of patients with measurements and median (interquartile range) of number of records for patients with measurements, number of children first diagnosed diabetes, **2013-2021**, over the first two years after the first prescription or diagnosis of mental health problem

|  | Monitoring period 2013-2021 | | | |
| --- | --- | --- | --- | --- |
|  | No antipsychotics | | On antipsychotics | |
|  | N (%) | Median (IQR) | N (%) | Median (IQR) |
| Total | 14989 |  | 1285 |  |
| Proportion of days on the drugs |  | N/A |  | 39 (8; 88) |
| Systolic BP | 2964 (19.8) | 2 (1; 3) | 701 (54.6) | 2 (1; 3) |
| Diastolic BP | 2958 (19.7) | 2 (1; 3) | 699 (54.4) | 2 (1; 3) |
| Pulse | 3841 (25.6) | 1 (1; 2) | 529 (41.2) | 1 (1; 2) |
| Height | 4980 (33.2) | 1 (1; 2) | 713 (55.5) | 2 (1; 3) |
| Weight | 5602 (37.4) | 1 (1; 2) | 789 (61.4) | 2 (1; 3) |
| BMI | 3294 (22.0) | 1 (1; 2) | 553 (43.0) | 1 (1; 2) |
| Total cholesterol | 182 (1.2) | 1 (1; 1) | 297 (23.1) | 1 (1; 2) |
| HDL | 165 (1.1) | 1 (1; 1) | 279 (21.7) | 1 (1; 2) |
| LDL | 129 (0.9) | 1 (1; 1) | 206 (16.0) | 1 (1; 2) |
| Glucose | 648 (4.3) | 1 (1; 1) | 339 (26.4) | 1 (1; 2) |
| HbA1c | 519 (3.5) | 1 (1; 1) | 213 (16.6) | 1 (1; 2) |
| Prolactin | 85 (0.6) | 1 (1; 1) | 311 (24.2) | 1 (1; 2) |
| Diagnosed with diabetes | 21 (0.1) |  | 10 (0.8) |  |

eTable 8 Number and proportion of patients with measurements **2013-2021**, over the first two years after the first prescription or diagnosis of mental health disorder, by metal health disorder

|  | Total N | Proportion of days on anti- psychotics,  median (IQR) | Heart  N (%) | Anthropo-metrics  N (%) | Lipids  N (%) | Blood glucose  N (%) | Prolactin  N (%) |
| --- | --- | --- | --- | --- | --- | --- | --- |
| **On antipsychotics** | | | |  |  |  |  |
| Psychosis | 77 | 57 (9; 93) | 58 (75.3) | 53 (68.8) | 29 (37.7) | 34 (44.2) | 24 (31.2) |
| Bipolar | 11 | 65 (14; 100) | 10 (90.9) | 9 (81.8) | 7 (63.6) | 7 (63.6) | 6 (54.5) |
| ASD | 476 | 44 (8; 92) | 255 (53.6) | 299 (62.8) | 112 (23.5) | 151 (31.7) | 116 (24.4) |
| Tourette | 94 | 37 (4; 83) | 55 (58.5) | 55 (58.5) | 17 (18.1) | 26 (27.7) | 19 (20.2) |
| No MH condition | 627 | 35 (8; 86) | 388 (61.9) | 392 (62.5) | 133 (21.2) | 214 (34.1) | 146 (23.3) |
| *Overall* | *1285* | *38 (8; 88)* | *766 (59.6)* | *808 (62.9)* | *298 (23.2)* | *432 (33.6)* | *311 (24.2)* |
|  |  |  |  |  |  |  |  |
| **No antipsychotics** | | | |  |  |  |  |
| Psychosis | 1452 | n/a | 631 (43.5) | 581 (40.0) | 52 (3.6) | 228 (15.7) | 34 (2.3) |
| Bipolar | 8 | n/a | 7 (87.5) | <5 | <5 | <5 | 0 |
| ASD | 11331 | n/a | 3705 (32.7) | 4394 (38.8) | 109 (1.0) | 627 (5.5) | 46 (0.4) |
| Tourette | 2198 | n/a | 652 (29.7) | 795 (36.2) | 20 (0.9) | 140 (6.4) | 5 (0.2) |
| *Overall* | *14989* | *n/a* | *4995 (33.3)* | *n/r* | *n/r* | *n/r* | *85 (0.6)* |

Heart measurements include: blood pressure; pulse. Anthropometrics: weight, BMI. Lipids: total cholesterol; HDL; LDL. Blood glucose: fasting glucose; HbA1C. n/a – not applicable. n/r – not reportable.
